# GSK3 inhibitors CHIR99021 and 6-bromoindirubin-3′-oxime inhibit microRNA maturation in mouse embryonic stem cells

**DOI:** 10.1038/srep08666

**Published:** 2015-03-02

**Authors:** Yongyan Wu, Fayang Liu, Yingying Liu, Xiaolei Liu, Zhiying Ai, Zekun Guo, Yong Zhang

**Affiliations:** 1 College of Veterinary Medicine, Northwest A&F University, Yangling 712100, Shaanxi, China; 2 Key Laboratory of Animal Biotechnology, Ministry of Agriculture, Northwest A&F University, Yangling 712100, Shaanxi, China; 3 College of Life Sciences, Northwest A&F University, Yangling 712100, Shaanxi, China

## Abstract

Wnt/β-catenin signalling plays a prominent role in maintaining self-renewal and pluripotency of mouse embryonic stem cells (mESCs). microRNAs (miRNAs) have critical roles in maintaining pluripotency and directing reprogramming. To investigate the effect of GSK3 inhibitors on miRNA expression, we analysed the miRNA expression profile of J1 mESCs in the absence or presence of CHIR99021 (CHIR) or 6-bromoindirubin-3′-oxime (BIO) by small RNA deep-sequencing. The results demonstrate that CHIR and BIO decrease mature miRNAs of most miRNA species, 90.4% and 98.1% of the differentially expressed miRNAs in BIO and CHIR treated cells were downregulated respectively. CHIR and BIO treatment leads to a slight upregulation of the primary transcripts of the miR-302–367 cluster and miR-181 family of miRNAs, these miRNAs are activated by Wnt/β-catenin signalling. However, the precursor and mature form of the miR-302–367 cluster and miR-181 family of miRNAs are downregulated by CHIR, suggesting CHIR inhibits maturation of primary miRNA. Western blot analysis shows that BIO and CHIR treatment leads to a reduction of the RNase III enzyme Drosha in the nucleus. These data suggest that BIO and CHIR inhibit miRNA maturation by disturbing nuclear localisation of Drosha. Results also show that BIO and CHIR induce miR-211 expression in J1 mESCs.

Embryonic stem cells (ESCs) and induced pluripotent stem (iPS) cells are attractive cell types in regenerative medicine because of their ability to self-renew and differentiate into all three germ layers^1^. Although the culture conditions needed to maintain pluripotency of ESCs has been established, the underlying molecular mechanism that regulates this pluripotency is not fully understood^2^. Studies focused on signal transduction pathways have provided new insights on the complex regulatory network underlying maintenance of pluripotency. The core pluripotency factors Oct4, Nanog, c-Myc, Sox2 and Klf4 have been found to play pivotal roles in sustaining pluripotency and preventing differentiation of ESCs^3^,^4^,^5^. Furthermore, these genes have been shown to act synergistically to reprogram fibroblasts into iPS cells^6^. Wnt/β-catenin signalling is critical for mouse ESC (mESC) self-renewal and pluripotency. Activation of Wnt/β-catenin signalling alleviates Tcf3 repression of pluripotency genes^7^. Moreover, β-catenin is able to enhance Oct4 activity and reinforce pluripotency in mESCs^8^. Taken together, Wnt/β-catenin signalling maintains pluripotency in mESCs by controlling the expression and transcriptional activity of core pluripotency factors.

miRNAs are single-stranded, non-coding RNAs that are 18–25 nucleotides in length. miRNAs regulate gene expression by binding to the 3′ untranslated region of target mRNAs and inducing mRNA degradation or inhibiting mRNA translation^9^. The biogenesis of miRNAs is well documented. Briefly, most of miRNA genes transcribed as long primary transcripts (pri-miRNA) by polymerase II, which are processed into mature miRNAs after nucleus and cytoplasmic processing. The microprocessor-complex consists of the RNase type III endonuclease Drosha, Di George syndrome critical region gene 8 (DGCR8) and additional co-factors recognize and cleave the pri-mRNA into ~70 nucleotide hairpin pre-miRNA^10^, and then the Exportin-5/Ran-GTP complex recognizes the pre-miRNA and exports pre-miRNA out of the nucleus. After entering the cytoplasm, the pre-miRNA is further processed by RNase III enzyme Dicer, the Dicer enzyme excises the pre-miRNA within the stem loop and yields the mature ~22–24 nucleotide miRNA-duplex^10^. There is a growing body of evidence that suggests that miRNAs play pivotal roles in the pluripotency and self-renewal of stem cells^11^,^12^. Several works reveal the global function of miRNAs in mESCs using cell lines deficient in Dicer or DGCR8^13^,^14^.

Small molecule inhibitors are emerging as important players in both the regulation of stem cell fate and in the reprograming of somatic cells. It has been shown that the leukaemia inhibitory factor (LIF)-2i medium that contains the mitogen-activated protein kinase inhibitor PD0325901, the glycogen synthase kinase 3 (GSK3) inhibitor CHIR and LIF is able to isolate and propagate pluripotent stem cells derived from mouse and other species^15^,^16^,^17^. Recent studies report that inhibition of GSK3 by CHIR, BIO or SB-216763 maintains self-renewal and pluripotency of mESCs^15^,^18^,^19^.

It is known that stabilisation of β-catenin and enhancement of adhesion is important for GSK3-inhibition-mediated mESC self-renewal and pluripotency^7^,^8^,^20^. However, whether maintenance of mESC pluripotency resulting from GSK3 inhibition is regulated by miRNAs is unknown. In this study, the gene expression of BIO treated J1 mESCs was investigated using microarray-based expression profiling. To understand miRNA changes in mESCs in response to GSK3 inhibition, small RNA deep-sequencing was employed. The results demonstrate that CHIR and BIO inhibit global maturation of miRNAs but upregulate miR-211.

## Results

### Activation of Wnt/β-catenin signalling promotes self-renewal and colony morphology of mouse pluripotent cells

**Figure 1:**
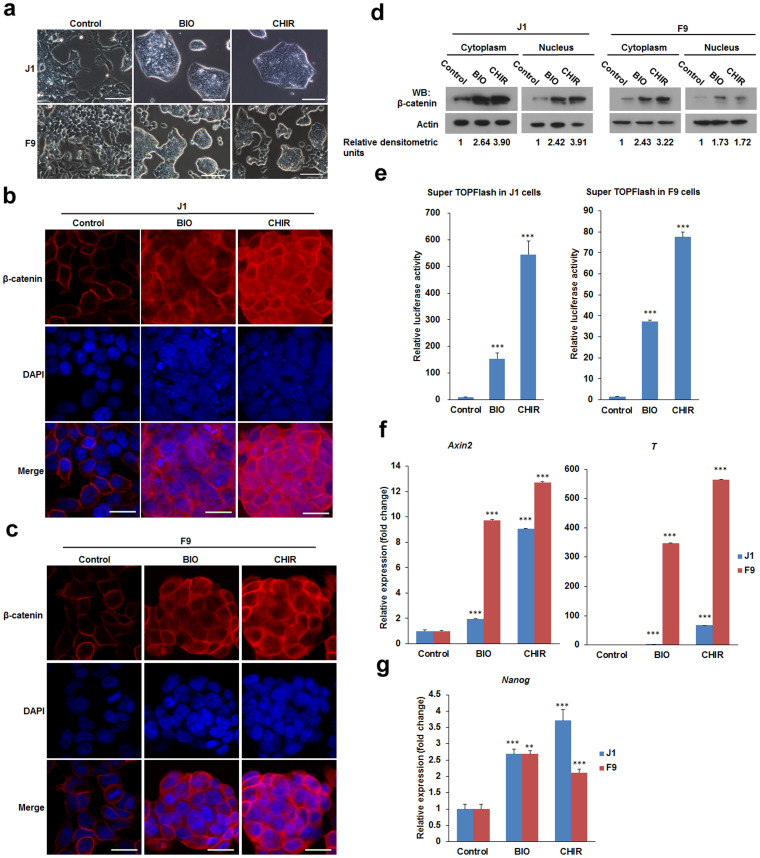
BIO and CHIR promote colony formation of J1 mESCs and F9 mEC cells. (a) J1 mESCs and F9 mEC cells were treated with 1 μM BIO or 3 μM CHIR for 24 h. Morphological changes were observed and recorded under a phase contrast microscope. Scale bar = 50 μm. (b) The expression and subcellular localisation of β-catenin in J1 mESCs treated with 1 μM BIO or 3 μM CHIR for 24 h was detected by immunofluorescence staining. Nuclei were stained with DAPI, scale bar = 20 μm. (c) The expression and subcellular localisation of β-catenin in F9 mEC cells treated with 1 μM BIO or 3 μM CHIR for 24 h was detected by immunofluorescence staining. Nuclei were stained with DAPI, scale bar = 20 μm. (d) Quantification of β-catenin by western blot. J1 mESCs or F9 mEC cells were treated with 1 μM BIO or 3 μM CHIR for 24 h. Cytoplasmic or nuclear β-catenin protein levels were quantitated by western blot and band densitometry was scanned by ImageJ and normalised to β-actin and the control. The relative densitometric data are shown below the immunoblots. (e) BIO and CHIR enhanced Wnt/β-catenin signalling. The activity of the β-catenin dependent reporter construct Super TOPFlash in J1 mESCs and F9 mEC cells treated with 1 μM BIO or 3 μM CHIR for 24 h were determined by luciferase reporter assays. The luciferase activity of Super TOPFlash was normalised to pTA-luc. (f) The expression of Wnt/β-Catenin signalling target genes Axin2 and T were determined by qPCR. (g) The expression of the pluripotency marker Nanog was determined by qPCR. Error bars indicate mean ± SD (n = 3), **, *p <* 0.01; ***, *p <* 0.001, compared with controls. Full-length blots are presented in Supplementary Figure 6.

It has been demonstrated that activation of Wnt/β-catenin signalling is able to maintain self-renewal and pluripotency of mESCs^8^. However, this is not true for human ESCs (hESCs). Activation of Wnt/β-catenin signalling in hESCs results in loss of self-renewal and induction of mesoderm lineage genes^21^. To determine the effect of Wnt/β-catenin signalling on self-renewal and morphology, J1 mESCs and F9 mouse embryonal carcinoma (mEC) cells were treated with the GSK3 inhibitors BIO and CHIR. We found that both J1 mESCs and F9 mEC cells grown in the presence of 1 μM BIO or 3 μM CHIR exhibited colony morphology and increased cell contacts. On the contrary, control cells were stretched and had few cell contacts (Fig. 1a).

To confirm that β-catenin protein was stabilised following BIO or CHIR treatment, immunofluorescence was used to determine subcellular localisation and immunoblots were used to determine protein levels. Immunofluorescence showed increased β-catenin in the cytoplasm and the nucleus of J1 mESCs and F9 mEC cells following BIO or CHIR treatment. By contrast, β-catenin mainly localised to the membrane in control cells (Fig. 1b and c). Immunoblots confirmed that BIO and CHIR treatment increased β-catenin in both the cytoplasm and the nucleus (Fig. 1d). Activation of the Wnt/β-catenin signalling was confirmed by luciferase reporter assay using a Super 7× TOPFlash reporter (Fig. 1e). Furthermore, the transcription of Wnt/β-catenin effectors *Axin2* and *T* was significantly upregulated by BIO or CHIR in J1 mESCs and F9 mEC cells (Fig. 1f).

Next, the expression of pluripotency genes was investigated following GSK3 inhibition. The relative mRNA expression level of *Nanog* was determined in response to BIO or CHIR in J1 mESCs and F9 mEC cells by qPCR. As expected, transcription of *Nanog* in J1 mESCs and F9 mEC cells was upregulated by both BIO and CHIR (Fig. 1g). Notably, although Wnt/β-catenin signalling was activated following BIO or CHIR treatment, the fold changes of reporter activity and downstream target genes (*Axin2 and T*) in F9 mEC cells showed significant differences from J1 mESCs in response to BIO or CHIR stimulation. This could be explained by intrinsic differences between the malignant (mEC) and non-malignant (mESC) pluripotent cells. Collectively, these data indicate that activation of Wnt/β-catenin signalling by BIO or CHIR promotes the expression of pluripotency associated genes.

### BIO regulates transcription factors and epigenetic regulators in mESCs

**Figure 2:**
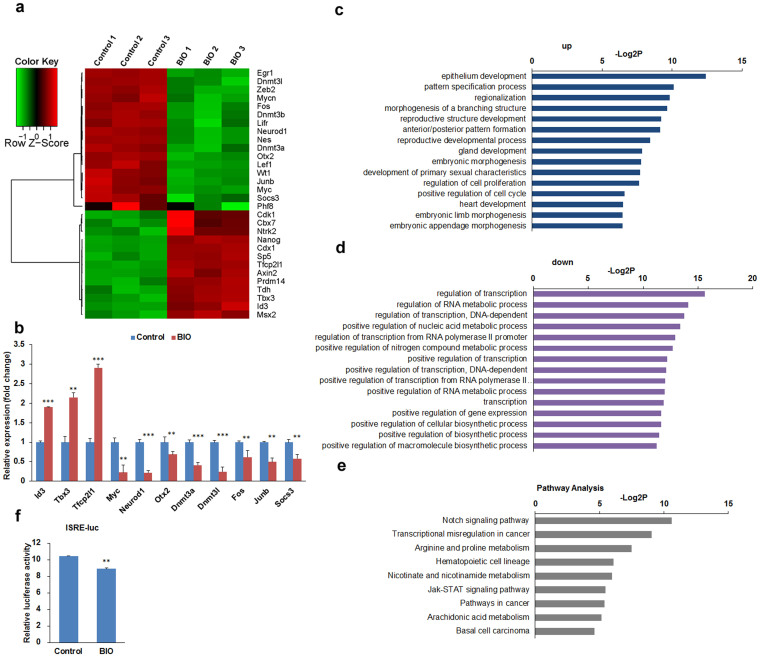
BIO regulates the expression of transcription factors and epigenetic regulators in mESCs. (a) Heatmap diagram of differentially expressed genes in J1 mESCs treated with 1 μM BIO. Differentially expressed genes were normalised by z-score, the pairwise distances of those genes were calculated by Pearson's correlation coefficient, and R (http://www.R-project.org) was employed to generate the heatmap. The cluster diagram represents 30 differentially expressed genes with fold change ≥1.5 and *p <* 0.05. Expression levels are shown as green for low intensities, and red for high intensities. (b) qPCR validation of the microarray data. The expression of genes was determined by qPCR. (c and d) GO term enrichment of the “biological process” category of BIO-regulated genes. Top 15 terms ranked according to the –Log2P of upregulated genes (c) or downregulated genes (d) were plotted. (e) KEGG pathway analysis of differentially expressed genes in BIO-treated J1 mESCs. (f) BIO inhibits the JAK-STAT signalling pathway. J1 mESCs were transfected with luciferase reporter pISRE-luc or pTA-luc for 24 h, then the cells were treated with 1 μM BIO or equal volume of DMSO for an additional 24 h. The luciferase activity of pISRE-luc was determined and normalised to pTA-luc. Error bars indicate mean ± SD (n = 3), **, *p <* 0.01; ***, *p <* 0.001, compared with controls.

To better understand the effects of BIO on transcription in ESC, genome-wide expression analysis was performed using microarray. mRNAs with fold changes greater than 1.5 and *p*-values less than 0.05 are presented in Supplementary Data 1. A total of 1398 differentially expressed genes were identified in BIO treated J1 mESCs compared with control-treated cells, of which 842 genes were upregulated and 556 were downregulated. The stem cell maintenance genes *Nanog*, *Tfcp2l1*, *Tbx3*, *Prdm14* and *Tdh* showed increased expression in BIO treated cells when compared with control cells (Fig. 2a). On the contrary, lineage-specific markers *Neurod1*, *Nes*, *Otx2* and *Wt1* were downregulated by BIO treatment (Fig. 2a). The expression of epigenetic regulators *Cbx7*, *Phf8*, *Dnmt3a*, *Dnmt3b* and *Dnmt3l* were altered by BIO (Fig. 2a). It should be noted that genes involved in JAK/STAT pathway, including *Socs3*, *Junb*, *Lifr* and *Myc*, were decreased following BIO treatment (Fig. 2a). Consistent with the microarray data, our qPCR analysis confirmed that BIO upregulates *Id3*, *Tbx3* and *Tfcp2l1*, and downregulates *Myc*, *Neurod1*, *Otx2*, *Dnmt3a*, *Dnmt3l*, *Phf8*, *Fos*, *Junb* and *Socs3* expression (Fig. 2b).

Functional annotation of differentially expressed genes by Gene Ontology (GO) revealed that BIO-upregulated genes were significantly enriched for terms linked to developmental processes, cell proliferation, cell cycle regulation and morphogenesis (Fig. 2c). BIO-downregulated genes were highly enriched for terms associated with metabolic processes, transcriptional regulation and biosynthetic processes (Fig. 2d). Kyoto Encyclopedia of Genes and Genomes (KEGG) pathway analysis showed that BIO-regulated genes are involved in the Notch signalling pathway, the JAK-STAT signalling pathway and metabolic processes (Fig. 2e). To validate the effect of BIO on the JAK-STAT pathway, the luciferase reporter assay was performed using the pISRE-luc plasmid that represent the JAK-STAT pathway. As shown in Figure 2f, BIO treatment decreased the luciferase activity of pISRE-luc significantly, confirming that BIO inhibits the JAK-STAT signalling pathway in J1 mESCs. Collectively, BIO treatment altered the expression of transcription factors and epigenetic regulators in J1 mESCs, fine-tuning the signalling pathways to maintain the stem cells characteristics.

### Small RNA deep-sequencing of BIO- and CHIR-treated mESCs

It has been shown that both Wnt/β-catenin signalling and miRNAs play pivotal roles in regulating self-renewal and differentiation of stem cells^2^, however, the expression pattern of miRNAs in ESCs after enhanced activation of Wnt/β-catenin signalling is not well known. To identify mature miRNAs expressed following treatment with GSK3 inhibitors, we sequenced miRNAs from J1 mESCs treated with BIO, CHIR, or DMSO (Control) using small RNA deep-sequencing technology (Supplementary Fig. 1). After removal of low-quality sequences, the 5′ and 3′ adapters, pollution reads and reads smaller than 18 nucleotides, 18,870,345 for the control-treated cells, 22,258,922 for the BIO-treated cells and 23,758,363 for the CHIR-treated cells of clean reads were extracted, respectively (Supplementary Data 2). The distribution of sequence lengths from these three small RNA libraries is presented in Supplementary Data 2, and small RNA annotation is shown in Supplementary Fig. 2. The clean reads were aligned to the GenBank non-coding RNA database (Supplementary Fig. 3) and the Rfam database (Supplementary Fig. 4) to find and remove non-coding RNA, such as rRNA, scRNA, tRNA, snRNA, snoRNA and other non-coding RNA. Subsequently, to find and remove degraded fragments of mRNA in the small RNA tags, small RNA reads were blasted against introns and exons of known mRNA (Supplementary Fig. 5). Finally, the clean reads were aligned to miRBase (Release 18) allowing only perfect matches.

After performing fold change analysis, we identified 157 miRNAs that had significant changes following BIO treatment when compared with the control library, in which 15 miRNAs were upregulated and 142 miRNAs were downregulated (fold change ≥1.5, *p<*0.05) (Supplementary Data 3). By contrast, CHIR upregulated 7 miRNAs and downregulated 366 miRNAs when compared with controls (fold change ≥1.5, *p <* 0.05) (Supplementary Data 4). Results show that the number of differentially expressed miRNAs following CHIR treatment is considerably more than BIO treated samples. This might be because of differences in specificity between the two inhibitors.

### BIO and CHIR differentially regulate the expression of ESC-specific cell cycle miRNAs

The expression and function of many miRNAs in pluripotent cells have been studied^22^. Interestingly, many pluripotency-maintaining miRNAs share a conserved seed sequence. These miRNAs are named ESC-specific cell cycle (ESCC) family of miRNAs^12^. The miR-302-367, miR-290-295, miR-17-92b, miR-106a-363 and miR-106b-25 cluster of miRNAs belong to the ESCC family of miRNAs^12^. The miR-302-367 cluster is comprised of miR-302b, miR-302c, miR-302a, miR-302d and miR-367, which are expressed specifically in pluripotent ESCs and mEC cells^23^. The miR-106a-363 cluster contains miR-106a, miR-18b, miR-20b, miR-19b, miR-92a and miR-363. Overexpression of miR-302-367 and miR-106a-363 clusters allows increases in iPS cell generation efficiency in mouse fibroblasts using three exogenous factors (Sox2, Klf4 and Oct4)^24^. The miR-290-295 cluster promotes pluripotency maintenance via regulating cell cycle phase distribution^25^.

**Figure 3:**
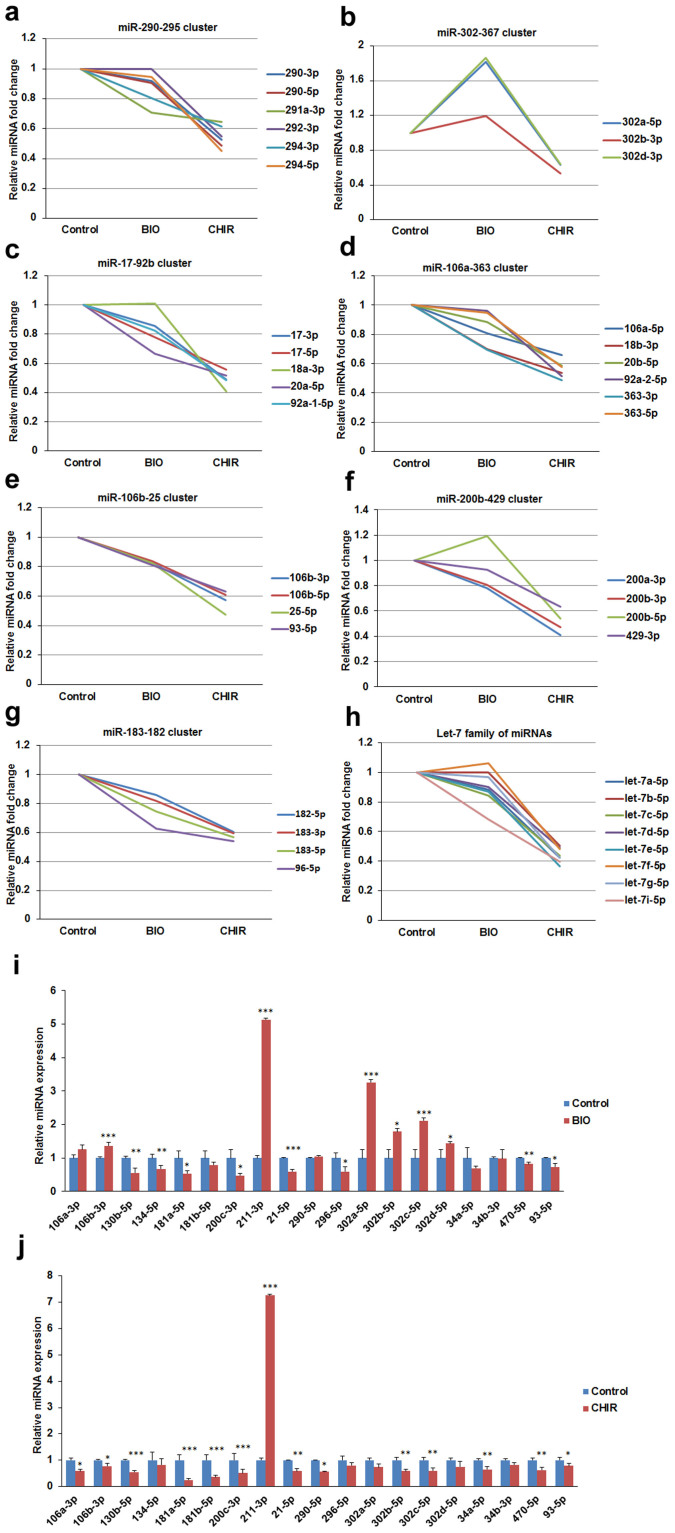
BIO and CHIR regulate the expression of the ESCC family of miRNAs in mESCs. (a–e) Relative fold change of mature ESCC family of miRNAs miR-290-295 cluster, miR-302-367 cluster, miR-17-92b cluster, miR-106a-363 cluster and miR-106b-25 cluster in control, BIO-, and CHIR-treated J1 mESCs detected by small RNA deep-sequencing. (f–h) Relative fold change of miR-200b-429, miR-183-182 cluster, and let-7 family in control, BIO- and CHIR-treated J1 mESCs detected by small RNA deep-sequencing. (i and j) qPCR validation of differentially expressed miRNA in BIO- or CHIR-treated J1 mESCs. J1 mESCs were treated with 1 μM BIO or 3 μM CHIR for 24 h, and then the expression of miRNAs was determined by qPCR. Error bars indicate mean ± SD (n = 3), *, *p <* 0.05; **, *p <* 0.01; ***, *p <* 0.001, compared with controls.

Our sequencing data showed that the expression of miR-302a and miR-302d were upregulated by BIO (Fig. 3b), but the other ESCC miRNAs were downregulated following BIO treatment although the fold change was less than 1.5 (Fig. 3a, 3c, 3d, 3e). By contrast, all of the ESCC miRNAs were downregulated by CHIR (Fig. 3a–e). The miR-200b-429 and miR-183-182 clusters, which are highly expressed in pluripotent cells, were downregulated by both BIO and CHIR (Fig. 3f, 3g). Furthermore, the let-7e family of miRNAs were significantly downregulated by CHIR treatment (Fig. 3h). Some of the miRNA changes were validated by qPCR. qPCR results confirmed that BIO upregulated miR-302a-5p, miR-302b-5p, miR-302c-5p and miR-302d-5p (Fig. 3i) and that CHIR significantly downregulated the expression of miR-290-5p, miR-106a-3p, miR-106b-3p, miR-302b-5p, miR-302c-5p, and miR-93-5p (Fig. 3j). It should be noted that miR-211 was strongly increased in both BIO- and CHIR-treated J1 mESCs, and that miR-181a was inhibited by both BIO and CHIR (Fig. 3i, 3j). These data show that BIO and CHIR differentially regulate miRNAs, where CHIR shows greater inhibitory effects on miRNA expression.

### GSK3 inhibitors impair processing of pri-miRNAs via disturbing distribution of Drosha

**Figure 4:**
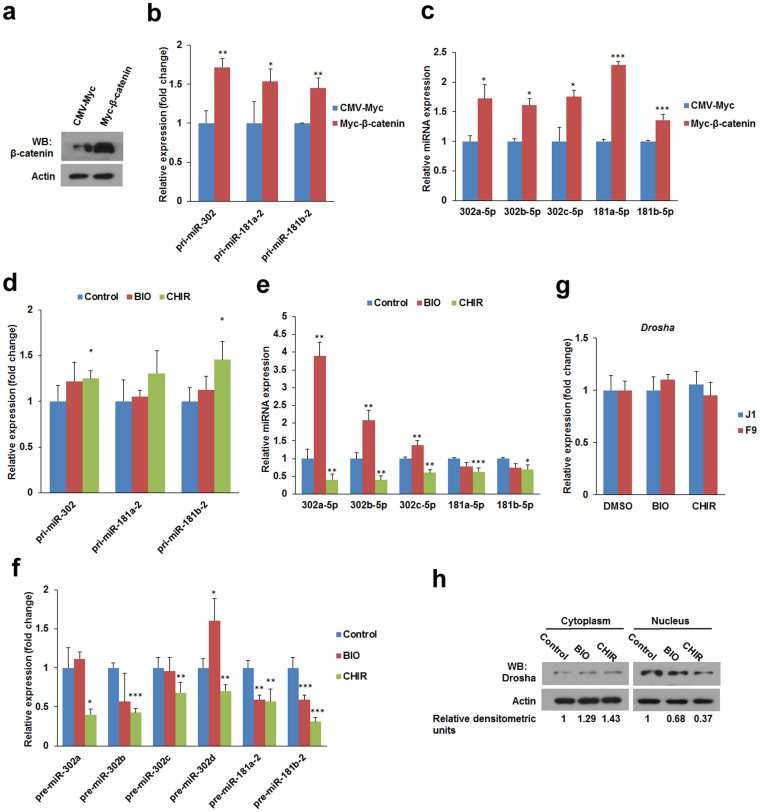
BIO and CHIR inhibit miRNA maturation in mESCs. (a) Overexpression of β-catenin in J1 mESCs. J1 mESCs were transfected with pCMV-Myc-β-catenin or empty vector pCMV-Myc for 48 h, then the expression of β-catenin was validated by western blot. (b) β-catenin promotes the expression of primary miRNAs of the miR-302-367 cluster and miR-181 family. The expression of pri-miR-302, pri-miR-181a-2, and pri-miR-181b-2 in β-catenin overexpressed J1 mESCs was determined by qPCR. (c) Overexpression of β-catenin upregulates mature miRNAs of the miR-302-367 cluster and miR-181 family. The expression of miR-302a-5p, miR-302b-5p, miR-302c-5p, miR-181a-5p, and miR-181b-5p in β-catenin transfected J1 mESCs was determined by qPCR. (d) Effect of GSK3 inhibitor on pri-miR-302, pri-miR-181a-2, and pri-miR-181b-2 expression. Transcription of pri-miR-302, pri-miR-181a-2, and pri-miR-181b-2 in BIO or CHIR-treated J1 mESCs was determined by qPCR. (e) CHIR downregulates mature miRNAs of the miR-302-367 cluster and miR-181 family. The expression of miR-302a-5p, miR-302b-5p, miR-302c-5p, miR-181a-5p, and miR-181b-5p in BIO- or CHIR-treated J1 mESCs was determined by qPCR. (f) The effect of BIO and CHIR on pre-miRNA expression in J1 mESCs. The expression of pre-miRNAs in BIO- or CHIR-treated J1 mESCs was determined by qPCR. (g) The effect of BIO and CHIR on Drosha expression. The expression of Drosha in J1 mESCs and F9 mEC cells after BIO or CHIR treatment was determined by qPCR. (h) GSK3 inhibitors disturbed the subcellular localisation of Drosha in J1 mESCs. J1 mESCs were treated with 1 μM BIO or 3 μM CHIR for 24 h. Drosha protein levels were detected in the cytoplasm and nucleus by western blot and quantitated by scanning densitometry using ImageJ. Bands were normalised to β-actin and the control. The relative densitometric data are shown below the immunoblots. Error bars indicate mean ± SD (n = 3), *, *p <* 0.05; **, *p <* 0.01; ***, *p <* 0.001, compared with controls. Full-length blots are presented in Supplementary Figure 7.

We noted that ~90% of miRNAs (142 out of 157) in the BIO-treated samples were downregulated (Supplementary Data 3) and that ~98% of miRNAs (367 of 373) in the CHIR-treated samples were downregulated (Supplementary Data 4). The expression of the miR-302-367 cluster and the miR-181 family of miRNAs are activated by Wnt/β-catenin pathway^26^,^27^, thereby we speculated that members of these family should be upregulated by BIO and CHIR because these inhibitors activate Wnt/β-catenin signalling. Unexpectedly, CHIR significantly downregulated the expression of miR-302-367 cluster and miR-181 family members, including miR-302a-5p, miR-302b-3p, miR-302d-3p, miR-181a-2-3p, miR-181a-5p, miR-181b-5p, miR-181c-5p, miR-181c-3p, and miR-181d-5p (Table 1). Additionally, BIO downregulated the expression of miR-181 family of miRNAs (Table 1). Results for miR-302a, miR-302b, miR-302c, miR-302d, miR-181a and miR-181b were validated by qPCR (Fig. 3i and 3j). β-catenin was overexpressed in J1 mESCs using the vector pCMV-Myc-*β-catenin* (Fig. 4a), and the expression of primary and mature forms of miR-302, miR-181a and miR-181b were determined by qPCR. The qPCR results showed that overexpression of β-catenin activates the transcription of pri-miR-302, pri-miR-181a-2 and pri-miR-181b-2 (Fig. 4b). Additionally, β-catenin overexpression increased the levels of mature miR-302a, miR-302b, miR-302c, miR-181a and miR-181b when compared with controls (Fig. 4c).

To better understand how BIO and CHIR regulate miRNAs that induced by Wnt/β-catenin signalling, we compared the expression of primary and mature miRNAs of miR-302-367 cluster and miR-181 family following BIO and CHIR treatment in J1 mESCs. qPCR results showed that BIO treatment resulted in a slight upregulation of pri-miR-302, pri-miR-181a-2 and pri-miR-181b-2. CHIR induced a higher level of expression of these primary miRNAs compared with BIO treatment (Fig. 4d). However, CHIR downregulated mature miR-302a, miR-302b, miR-302c, miR-181a and miR-181b expression, and BIO slightly downregulated miR-181a and miR-181b (Fig. 4e). The results indicate that BIO and CHIR inhibit the processing of primary miRNAs. To further analyse this, the precursor forms of miR-302a, miR-302b, miR-302c, miR-302d, miR-181a and miR-181b was examined by qPCR in J1 mESCs. Consistently, pre-miR-302a, pre-miR-302b, pre-miR-302c and pre-miR-302d were reduced following CHIR treatment, and pre-miR-181a-2 and pre-miR-181b-2 were downregulated by both BIO and CHIR (Fig. 4f).

The RNase III enzyme Drosha and its essential cofactors mediate nuclear processing of pri-miRNA^28^. The expression of Drosha was tested following treatment with BIO or CHIR in J1 mESCs and F9 mEC cells. The results suggest that BIO and CHIR do not affect Drosha expression (Fig. 4g). Recent studies show that phosphorylation of Drosha at S300 and S302 by GSK3β is required for its nuclear localisation^29^. We investigated the distribution of Drosha by immunoblot. The results demonstrated that either BIO or CHIR treatment leads to a reduction of Drosha protein in the nucleus (Fig. 4h). These data suggest that BIO and CHIR inhibit miRNA maturation, particularly inhibiting maturation of Wnt/β-catenin signalling-activated miR-302-367 cluster and miR-181 family of miRNAs, is probably because inhibition of GSK3 activity disturbs the nuclear localisation of Drosha.

### GSK3 inhibitors and β-catenin induce the expression of miR-211

**Figure 5:**
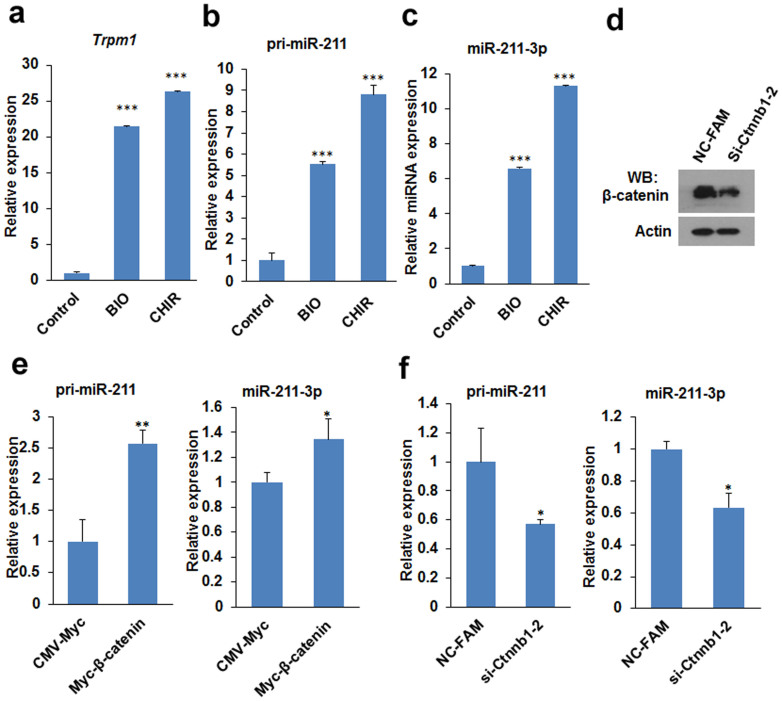
Activation of Wnt/β-catenin signalling induces miR-211 expression in mESCs. J1 mESCs were treated with 1 μM BIO, 3 μM CHIR or equal volume of DMSO (control) for 24 h, the transcription levels of Trpm1 (a), pri-miR-211 (b) and mature form miR-211 (c) were determined by qPCR. (d) Knockdown of β-catenin in J1 mESCs. J1 mESCs were transfected with siRNAs targeted against β-catenin (si-Ctnnb1-2) or negative control siRNAs (NC-FAM) for 48 h, and then β-catenin protein level was determined by western blot. (e) Overexpression of β-catenin induced primary and mature miR-211 expression. The expression of primary and mature miR-211 in β-catenin overexpressed J1 mESCs was determined by qPCR. (f) Knockdown of β-catenin repressed miR-211 expression. The expression of primary and mature miR-211 in β-catenin knockdown J1 mESCs was determined by qPCR. Error bars indicate mean ± SD (n = 3), *, *p <* 0.05; **, *p <* 0.01; ***, *p <* 0.001, compared with controls. Full-length blots are presented in Supplementary Figure 8.

The sequencing data shows that miR-211 is dramatically upregulated in response to BIO and CHIR treatment (Supplementary Data 3 and 4) even though most other miRNAs are downregulated. Mouse miR-211 is located on chromosome 7 and embedded in intron 2 of the transient receptor potential cation channel subfamily member 1 (*Trpm1*) gene, indicating that miR-211 might be expressed similarly to *Trpm1*. The microarray data showed that BIO induces *Trpm1* expression in J1 mESCs (Supplementary Data 1). The qPCR results further confirmed that both *Trpm1* and pri-miR-211 were upregulated following either BIO or CHIR treatment (Fig. 5a and b). Consistently, the mature form of miR-211 was significantly upregulated by BIO and CHIR treatment (Fig. 5c). Since BIO and CHIR are able to activate Wnt/β-catenin signalling by stabilising β-catenin, we wanted to determine if either BIO- or CHIR-upregulated miR-211 is mediated directly by β-catenin. Therefore, we analysed the expression of both primary and mature forms of miR-211 under varying levels of β-catenin. J1 mESCs were transfected with pCMV-Myc-*β-catenin* or siRNAs targeting β-catenin and the expression of β-catenin was determined by immunoblot. The results showed that overexpression of β-catenin increased β-catenin level (Fig. 4a), and RNAi decreased β-catenin expression (Fig. 5d). qPCR results showed that overexpression of β-catenin significantly upregulates pri-miR-211 and miR-211-3p in J1 mESCs (Fig. 5e). Furthermore, knockdown of β-catenin led to a reduction of both primary and mature miR-211 (Fig. 5f). These data reveal that β-catenin increases the expression of miR-211 and that activation of Wnt/β-catenin signalling by GSK3 inhibitors induces miR-211 expression.

## Discussion

Studies reveal that both miRNAs and small molecule inhibitors have big roles in maintaining pluripotency and in reprogramming processes^30^,^31^,^32^,^33^. More recently, the GSK3 inhibitor has been shown to promote self-renewal and pluripotency of mESCs^15^,^18^,^19^. We previously showed that the GSK3 inhibitor CHIR promotes self-renewal by regulating pluripotency factors, epigenetic regulators, and long non-coding RNAs^34^. In this study, we analysed the effect of the GSK3 inhibitors BIO and CHIR on miRNAs expression in J1 mESCs.

Our results demonstrate that both BIO and CHIR are able to enhance colony morphology of J1 mESCs and F9 mEC cells. Stabilisation of β-catenin is critical for GSK3 inhibitor-mediated stem cell self-renewal and pluripotency^35^,^36^. However, our previous work revealed that some CHIR-responsive genes do not respond to β-catenin and that CHIR also influences pathways other than the Wnt/β-catenin signalling pathway^34^. This suggests that GSK3 inhibitor-mediated self-renewal and pluripotency may not depend solely on Wnt/β-catenin signalling. To better understand events that occur downstream of GSK3 inhibition, microarray was performed following treatment with BIO. Like CHIR, BIO enhanced the expression of pluripotency markers and inhibited the expression of *Dnmt3a*, *Dnmt3b*, and *Dnmt3l*. This indicates that the expression of pluripotency genes and global DNA hypomethylation is important for GSK3 inhibition-mediated self-renewal and pluripotency^37^. Prior studies demonstrate that BIO inhibits JAK/STAT3 signalling in human melanoma cells^38^. Our data show that the downstream targets of JAK-STAT signalling are downregulated by BIO. These results suggest that BIO mediates self-renewal and pluripotency through the Wnt/β-catenin signalling pathway and through other signalling pathways.

The small RNA deep-sequencing data shows that most of differentially expressed miRNAs in the BIO- and CHIR-treated cells were downregulated, including the Wnt/β-catenin-regulated miR-302-367 cluster and miR-181 family members. However, the pri-miR-302, pri-miR-181a-2 and pri-miR-181b-2 show upregulation in BIO- and CHIR-treated cells, this indicates that the reduced expression of mature miRNAs might be because of the inhibition of pri-miRNAs processing, rather than inhibition of miRNA transcription. The qPCR results of precursor form of the miR-302-367 cluster and miR-181 family members confirmed this notion. The RNaseIII enzyme Drosha is a key factor in miRNA biogenesis. Drosha cleaves pri-miRNA transcripts in the nucleus to generate pre-miRNA. It has been shown that phosphorylation of Drosha by GSK3β is critical for the nuclear localisation of Drosha, and that inhibiting the phosphorylation leads to cytoplasmic localisation of Drosha^29^,^39^. Our data demonstrate that BIO and CHIR treatment do not affect Drosha expression but instead decrease the nuclear accumulation of Drosha in J1 mESCs. Hence, we speculated that BIO and CHIR inhibit maturation of miRNA by inhibiting the phosphorylation of Drosha and blocking its entry into nucleus. This would prevent processing of pri-miRNA.

The ESCC family of miRNAs play critical roles in maintaining pluripotency. We found that the expression levels of the ESCC family of miRNAs were lower in CHIR-treated samples compared with BIO-treated samples. Specifically, the miR-290-295 cluster, miR-302-367 cluster, miR-17-92b cluster, miR-106a-363 cluster, and miR-106b-25 cluster all showed decreased expression. In addition, CHIR treatment inhibited more miRNAs than BIO treatment. Both BIO and CHIR are selective inhibitors of GSK3α and GSK3β, however, it has been shown that CHIR has a higher affinity for GSK3β^40^. We hypothesise that CHIR may be a stronger inhibitor of Drosha phosphorylation thus greatly altering the localisation of Drosha and inhibiting miRNA maturation.

Gene expression profiling and chromatin immunoprecipitation coupled high-throughput DNA sequencing identified downstream target genes of the Wnt/β-catenin signalling pathway. The data show that β-catenin affects the expression of a large number of genes by binding to regulatory elements containing classic Lef/Tcf motifs^41^,^42^. miRNAs play a pivotal role in determining stem cell fate and are becoming an area of intense interest in stem cell biology. The relationship between Wnt/β-catenin signalling and miRNAs is complex; β-catenin activates miRNAs expression^26^,^27^,^43^, and in turn, Wnt/β-catenin signalling is regulated by miRNAs^44^,^45^. Our data demonstrate that overexpression of β-catenin activates miR-211, and that activation of Wnt/β-catenin signalling by BIO and CHIR upregulates miR-211 expression. Further investigation is needed to identify miR-211 target genes and their function in ESCs. It should be noted that not all miRNA species in BIO- and CHIR-treated samples were downregulated. Specifically, 15 miRNAs were upregulated in BIO-treated samples and 7 miRNAs were upregulated in CHIR-treated samples. A possible explanation is that other mechanisms are involved in regulating Drosha localisation in addition to GSK3β-mediated phosphorylation. Also, substrate preference may exist in the processing of pri-miRNA and certain miRNA species are better substrates for enzyme^29^.

In conclusion, using small RNA deep-sequencing technology, we compared the expression of mature miRNAs in J1 mESCs treated with GSK3 inhibitors. We found that inhibition of GSK3 by BIO or CHIR suppressed a large number of miRNAs, including Wnt/β-catenin signalling activated miRNAs. However, the qPCR data showed that BIO and CHIR did not inhibit transcription of Wnt/β-catenin-regulated pri-miRNAs. This suggests that GSK3 inhibitors impair the processing of pri-miRNAs. Furthermore, the results demonstrate that activation of Wnt/β-catenin signalling by BIO or CHIR induces miR-211 expression. This study reveals the role of GSK3 inhibitors in the regulation of miRNA expression in mESCs and provides reference data for further studies.

## Methods

### Reagents

CHIR99021 and rabbit anti-β-catenin antibody were purchased from Santa Cruz Biotechnology (Santa Cruz, CA). BIO was purchased from Sigma-Aldrich. β-Actin mouse monoclonal antibody was purchased from Beijing TransGen Biotech Co., Ltd (Beijing, China). Drosha antibody was purchased from Cell signaling (Danvers, MA). Alexa Fluor 555-labeled goat anti-rabbit IgG and anti-rabbit/mouse horseradish peroxidase-conjugated secondary antibody were obtained from the Beyotime Institute of Biotechnology (Jiangsu, China).

### Cell culture

The J1 mESC line purchased from the American Type Culture Collection (Manassas, VA) was grown on 0.1% (w/v) gelatin-coated tissue culture plates without feeders in mESC medium [Knockout DMEM supplemented with 15% (v/v) Knockout Serum Replacement, 1× non-essential amino acids, 100 μM β-mercaptoethanol, 2 mM glutamine, 50 U/ml penicillin, 50 μg/ml streptomycin (Life Technologies, Grand Island, NY) and 1000 U/ml LIF (ESGRO, Millipore, MA)]. F9 mEC cells were purchased from the cell bank of the Chinese Academy of Sciences and maintained in 0.1% gelatin-coated plates with Dulbecco's modified Eagle's medium supplemented with 10% fetal bovine serum.

### Luciferase reporter assays

The pTA-luc plasmid was purchased from Clontech (Mountain View, CA). The Super 7× TOPFlash luciferase reporter was constructed as described previously^34^. Pathway reporter pISRE-luc was purchased from the Beyotime Institute of Biotechnology. J1 mESCs or F9 mEC cells were cotransfected with luciferase reporter and the *Renilla* luciferase plasmid pRL-SV40 (Promega) using Lipofectamine 2000 Reagent (Invitrogen). After 24 h, transfected cells were treated with 1 μM BIO, 3 μM CHIR and equal volume of DMSO respectively for an additional 24 h. Luciferase assays were performed using a Dual-Luciferase Reporter Assay System (Promega, Madison, WI) according to the manufacturer's instructions.

### RNA isolation, microarray-based gene expression profiling and small RNA deep-sequencing

CHIR and BIO were dissolved in DMSO at a final concentration of 10 mM. At 1 d before treatment, J1 mESCs or F9 mEC cells were seeded on a gelatin-coated 6-well plate to reach 30–50% confluence at the time of treatment. BIO and CHIR were added to medium at a final concentration of 1 μM and 3 μM respectively, an equal volume of DMSO was added to medium for control cells. For each treatment (control or inhibitor treated), three independent experiments were conducted to prepare the samples. At 24 h after treatment, total RNA was extracted using Trizol reagent (Life technologies, Carlsbad, CA) following the manufacturer's instructions. RNA integrity was checked by an Agilent Bioanalyzer 2100 system (Agilent technologies, Santa Clara, CA). Qualified total RNA of each sample was divided into two copies, one for microarray experiment and the other one for Small RNA deep-sequencing. The microarray experiment was performed as described previously^34^.

For Small RNA sequencing, the total RNA from three independent experiments of each treatment was pooled respectively. Small RNA library construction and sequencing was performed by Beijing Genomics Institute (Shenzhen, China). Briefly, sRNA (18 to 30 nt) was gel purified and ligated to the 3′ and 5′ adaptor. The ligated products were reverse transcribed, followed by acrylamide gel purification and PCR amplification to generate sRNA libraries^46^. The library was loaded on an Agilent 2100 Bioanalyzer system to check size, purity and concentration. Libraries were sequenced on an Illumina HiSeq 2000 sequencing system (Illumina, San Diego, CA). Sequencing data has been submitted to the Gene Expression Omnibus (GEO) (accession ID: GSE54145).

### Bioinformatics analysis of sequencing data

Low quality sequences and adaptors were removed from raw small RNA sequence data. The 18–30 nt clean small RNAs were aligned to the miRNA precursors of *Mus musculus* (mature miRNA if there is no precursor information in miRBase18). To measure miRNA expression with high confidence, only perfectly matched sequences were considered to be conserved miRNAs. The expression of miRNAs in the two samples (control and treatment) was normalized to one million by the total number of miRNAs in each sample (Normalization formula: normalized expression = actual miRNA count/total count of clean reads*1,000,000). Following normalization, the fold change between treatment and control sample was calculated as: fold change = log2 (treatment/control), the p-value was calculated using the formula described previously^47^. If the miRNA expression was zero, then it was revised to 0.01, if the miRNA gene expression of two samples was less than 1, they did not participate in analysis of differential expression.

### qPCR analysis

The miRNAs expression was validated by poly (A)-tailed qPCR. Total RNA was extracted from small molecules treated or control sample using Trizol reagent, 1 μg of RNA was reverse-transcribed to cDNA using miScript II RT Kit (Qiagen GmbH, Hilden, Germany) according to the manufacturer's instructions. For qPCR experiments of mRNAs, reverse-transcription was performed using a SYBR PrimeScript RT reagent Kit (Perfect Real Time) (Takara, Dalian, China). qPCR was performed using SYBR Premix Ex Taq II (Takara, Dalian, China) on an StepOne Plus PCR system (Applied Biosystems, Foster City, CA). All primers used are provided in Supplementary Table 1. The specificity of the primer amplicons was examined by the analysis of a melting curve. The relative expression of miRNA was normalized to small nuclear RNA (Rnu6) expression and relative to the control. The relative expression of mRNA was normalized to *GAPDH* expression and relative to the control. Data were expressed as the fold change = 2^−ΔΔCt^.

### Gene ontology (GO) and KEGG pathway analysis

Data screening was carried out based on a gene expression fold change of ≥1.5 and statistical significance of *p* < 0.05. Biological themes of the differentially expressed genes were identified by the biological processes of GO categories using the online tool of the Database for Annotation, Visualization, and Integrated Discovery (DAVID)^48^. KEGG pathway analysis was performed using the SAS online program (http://www.ebioservice.com/eng/index.asp) with a hit number of ≥5 and enrichment test p-value of <0.05.

### Overexpression and knockdown of β-catenin

To construct the expression vector pCMV-Myc-β-catenin, ORF sequence of β-catenin was amplified by RT-PCR and then inserted into the pCMV-Myc vector. The sequences of small interference RNA (siRNA) target β-catenin and negative control were described previously^34^. J1 mESCs were transfected with pCMV-Myc-β-catenin or siRNAs using Lipofectamine™ 2000 Reagent (Invitrogen) for 48 h, then the total RNA was extracted using Trizol reagent and total protein was extracted using RIPA buffer (Thermo scientific, MA).

### Subcellular fractionation

Control or GSK3 inhibitor treated cells were harvested, washed with PBS and pelleted. Nuclear and cytoplasmic proteins were prepared using NE-PER Nuclear and Cytoplasmic Extraction kit (Thermo scientific) according to the manufacturer's instructions. Nuclear and cytoplasmic proteins were separated by SDS-PAGE and analysed by western blot.

### Western blot analysis

Total proteins were separated on 12% acrylamide gels and transferred to PVDF membranes (Millipore, Bedford, MA) for 2.5 h at 100 V. Membranes were blocked in 5% non-fat milk/TBST for 2 h and then incubated with the primary antibody overnight at 4°C. Membranes were washed three times with TBST and then incubated with the secondary antibody for 2 h, followed by wash three times for 10 min. The immunoblots were developed by using SuperSignal west pico chemiluminescent substrate (Thermo scientific).

### Immunofluorescence staining

J1 mESCs or F9 mEC cells were fixed and permeabilized using immunostaining fixation buffer and then blocked in blocking buffer (Beyotime Institute of Biotechnology). Subsequently, cells were incubated with the indicated primary antibody overnight at 4°C, followed by three washes with washing buffer (Beyotime Institute of Biotechnology) for 5 min and then incubation with an Alexa Fluor 555-conjugated secondary antibody for 2 h at room temperature. Nuclei were stained with DAPI. Cells were photographed under an inverted fluorescence microscope (Nikon, Tokyo, Japan).

### Statistical analysis

Data are reported as the mean ± standard deviation (SD), and analysed using the Student's t-test. A value of *p* < 0.05 was considered significant.

## Author Contributions

W.Y., L.F., L.Y. and L.X. carried out the experiment. W.Y., G.Z., L.X. analysed the data. A.Z. performed the statistical analysis. W.Y. and G.Z. drafted the manuscript. G.Z. and Z.Y. proposed and supervised the research. All authors read and approved the final manuscript.

## Supplementary Material

Supplementary Information Supplementary Figures, Table and legends

Supplementary Information Dataset 1

Supplementary Information Dataset 2

Supplementary Information Dataset 3

Supplementary Information Dataset 4

## Figures and Tables

**Table 1:** Expression of miR-302-367 cluster and miR-181 family members in BIO and CHIR treated mESCs detected by small RNA deep-sequencing

	Expressed reads			Normalized reads			Fold change	
miRNA	Control	BIO	CHIR	Control	BIO	CHIR	BIO vs. Control	CHIR vs. Control
miR-302a-5p	171	366	136	9.06	16.44	5.72	0.86	−0.66
miR-302b-3p	27	38	18	1.43	1.71	0.76	0.25	−0.92
miR-302d-3p	46	101	37	2.44	4.54	1.56	0.90	−0.65
miR-181a-2-3p	84	39	13	4.45	1.75	0.55	−1.35	−3.02
miR-181a-5p	991	710	216	52.52	31.90	9.09	−0.72	−2.53
miR-181b-5p	1459	1008	344	77.32	45.29	14.48	−0.77	−2.42
miR-181c-3p	685	386	410	36.30	17.34	17.26	−1.07	−1.07
miR-181c-5p	2611	2161	1886	138.37	97.08	79.38	−0.51	−0.80
miR-181d-5p	44939	50082	36427	2381.46	2249.97	1533.23	−0.08	−0.64
