# Supplementary material for: GSK3 inhibitors CHIR99021 and 6-bromoindirubin-3′-oxime inhibit microRNA maturation in mouse embryonic stem cells
Source: Sci Rep. 2015 Mar 2;5:8666. doi: 10.1038/srep08666 (PMC4345320; doi:10.1038/srep08666)
Supplement: Supplementary Information — Supplementary Figures, Table and legends [file srep08666-s1.pdf]

GSK3 inhibitors CHIR99021 and 6-bromoindirubin-3'-oxime inhibit microRNA maturation in mouse embryonic stem cells

Yongyan Wu 1,2, Fayang Liu 1,2, Yingying Liu 2,3, Xiaolei Liu 2,3, Zhiying Ai 2,3, Zekun Guo 1,2,*, Yong Zhang 1,2,*

1 College of Veterinary Medicine, Northwest A&F University, Yangling 712100, Shaanxi, China

2 Key Laboratory of Animal Biotechnology, Ministry of Agriculture, Northwest A&F University, Yangling 712100, Shaanxi, China

3 College of Life Sciences, Northwest A&F University, Yangling 712100, Shaanxi, China

Supplementary Figure 1. **Experimental scheme for sample preparation of small RNA deep-sequencing and microarray analysis.**

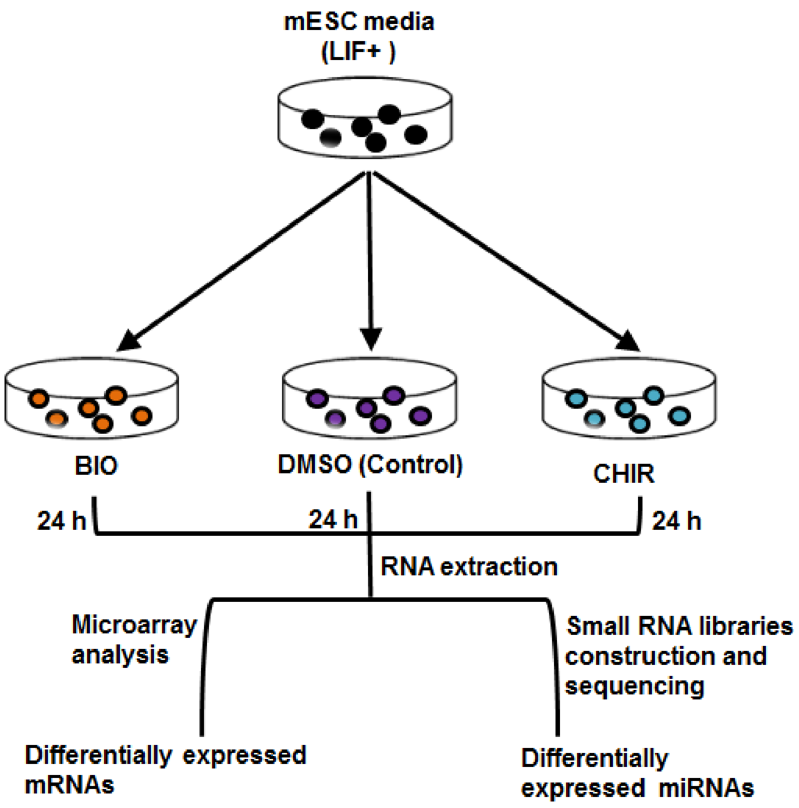

Supplementary Figure 2. **Annotation of sequenced small RNAs from Control, BIO and CHIR libraries.**

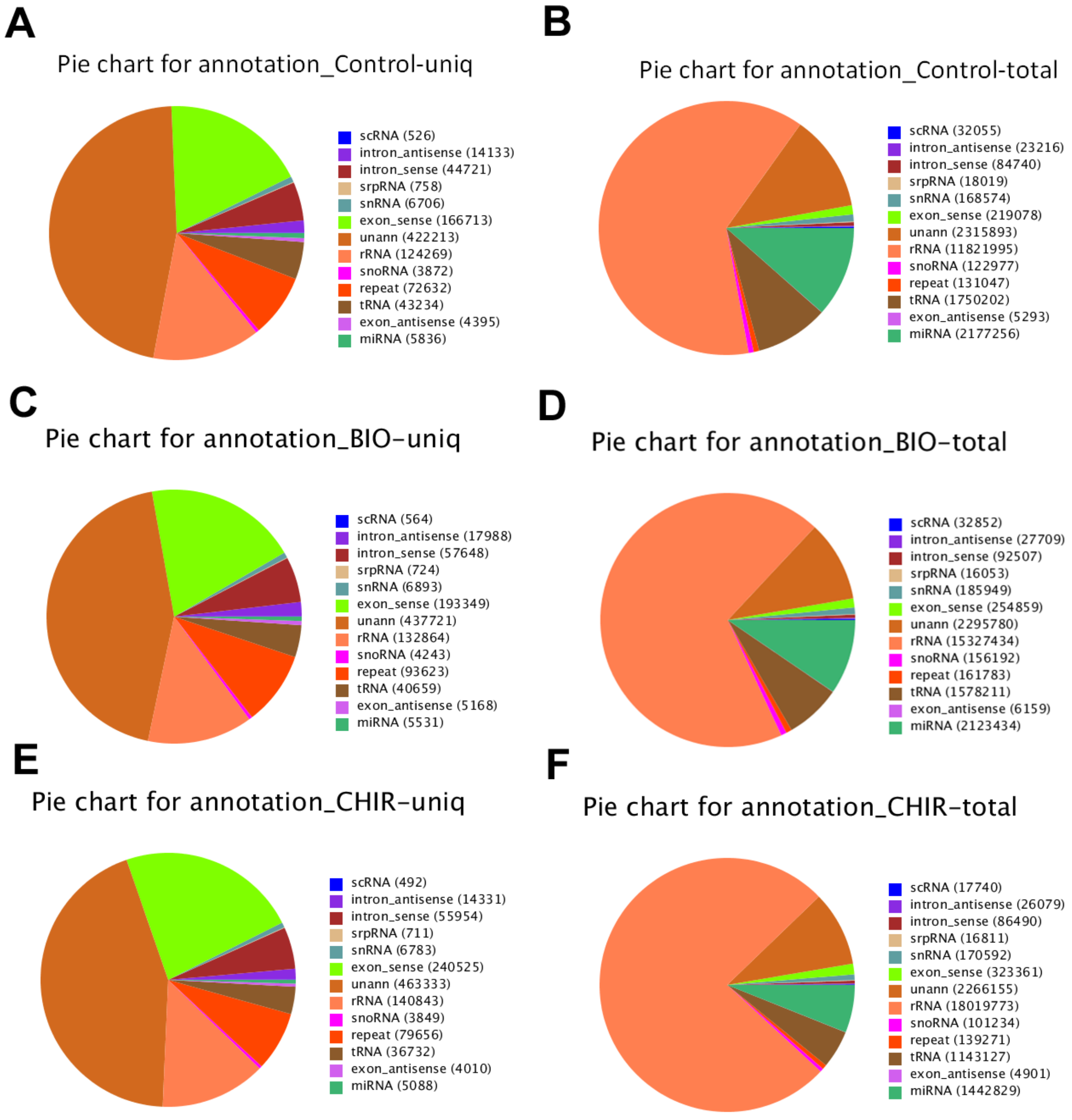

Supplementary Figure 3. **Alignment of sequenced small RNAs to GenBank non-coding RNA database.**

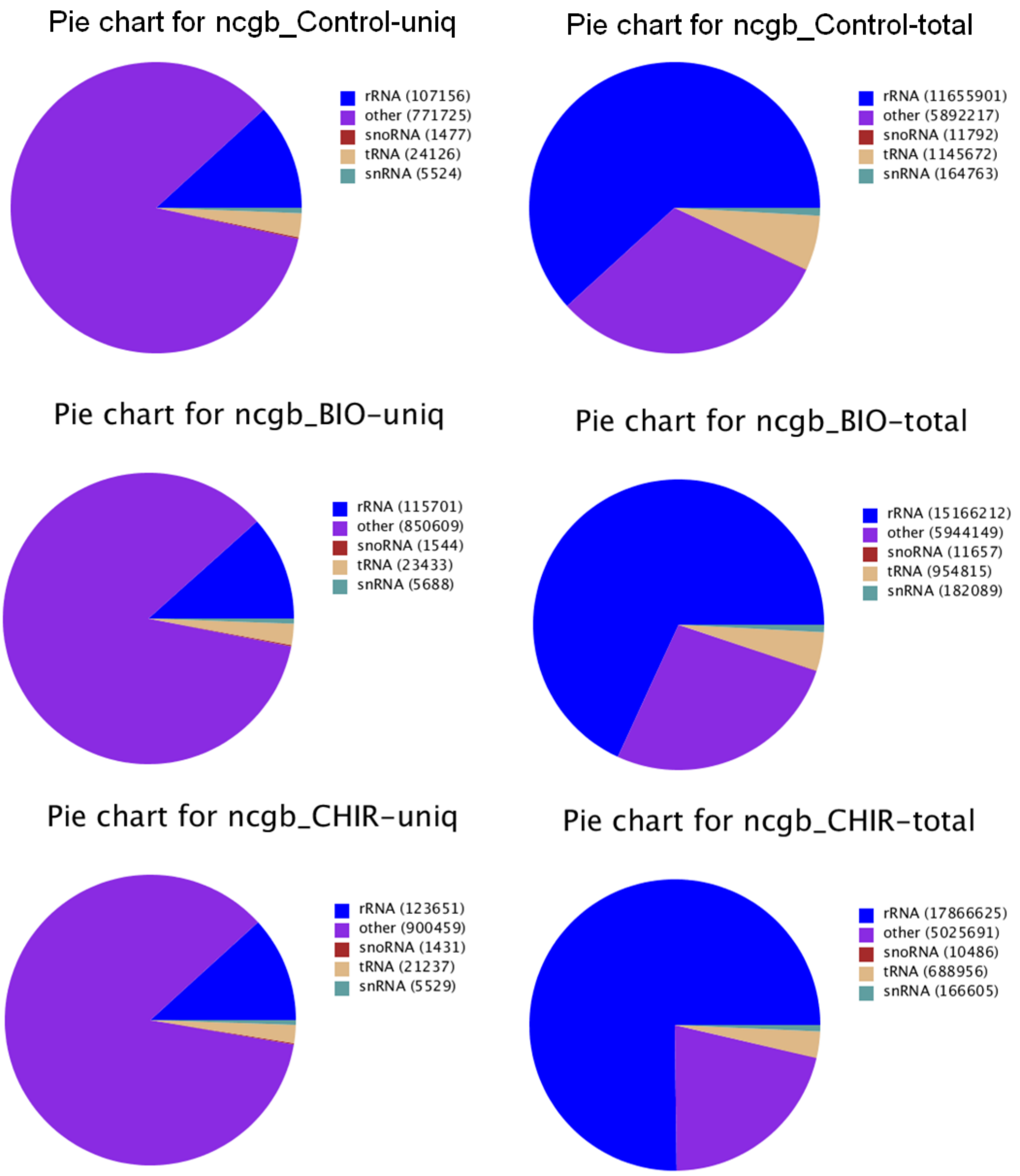

Supplementary Figure 4. **Alignment of sequenced small RNAs to the Rfam database.**

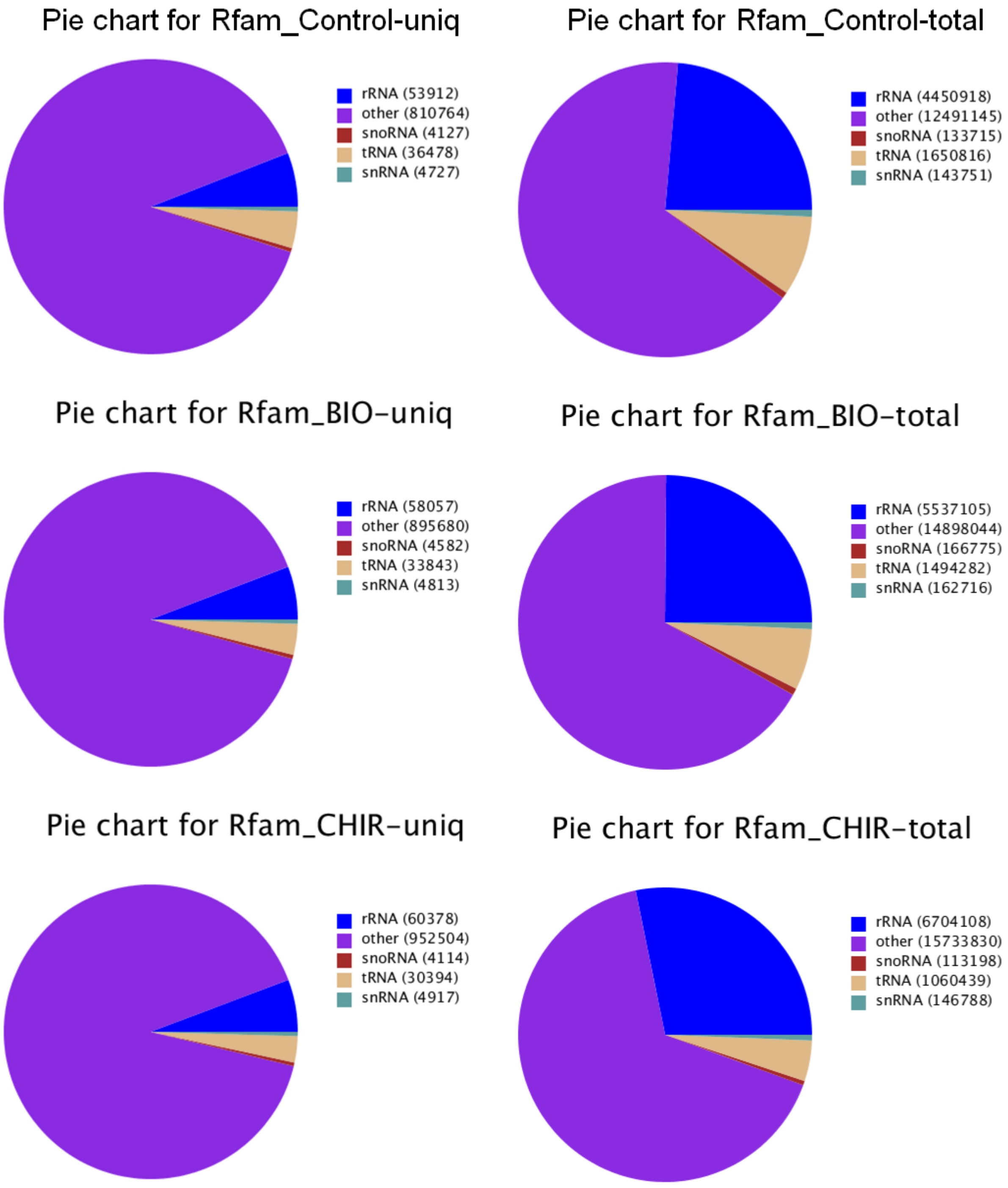

Supplementary Figure 5. **Alignment of sequenced small RNAs to introns and exons of mRNA.**

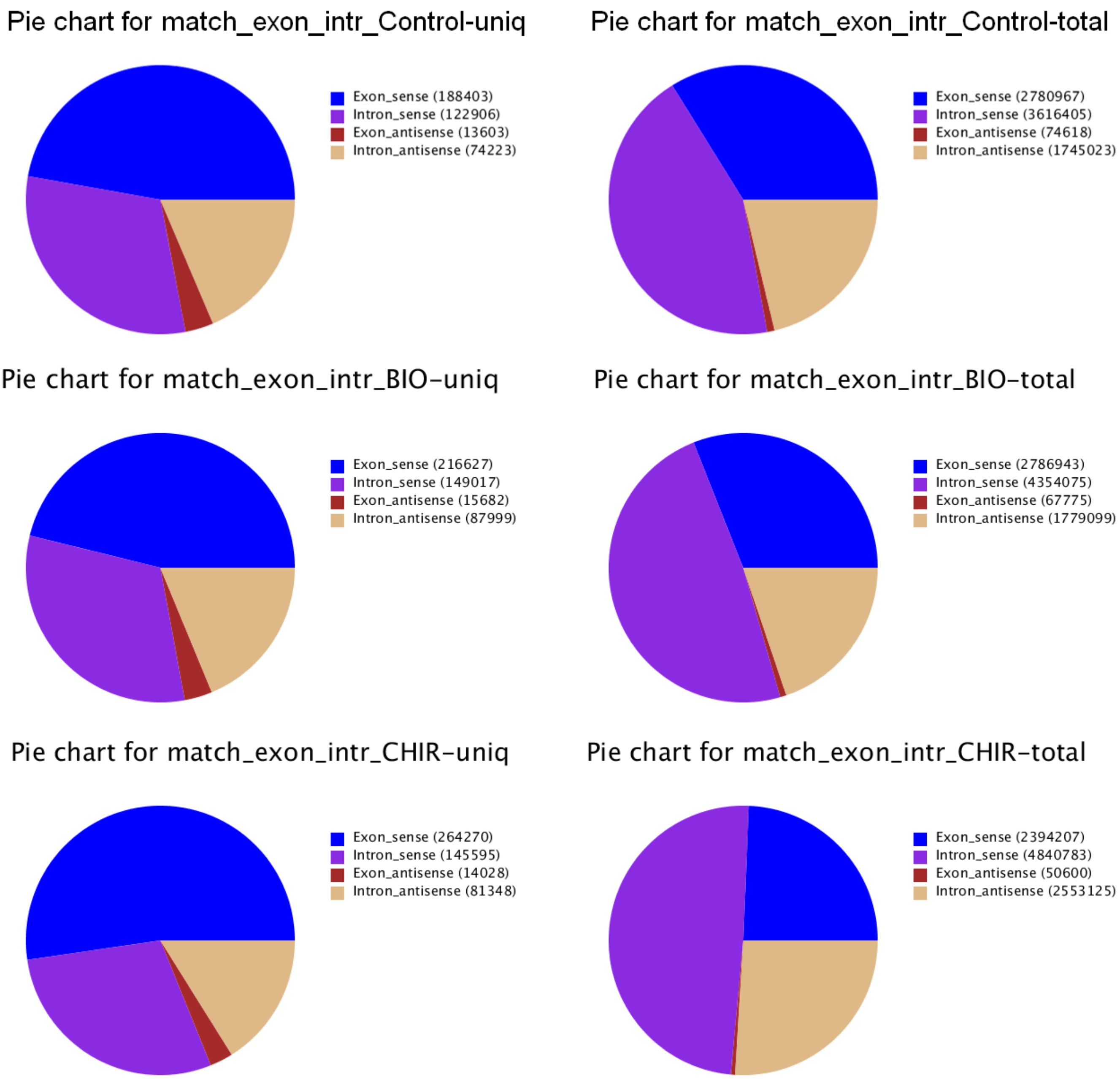

Supplementary Figure 6. **Full-length blots of Figure 1 (d) in the main text.**

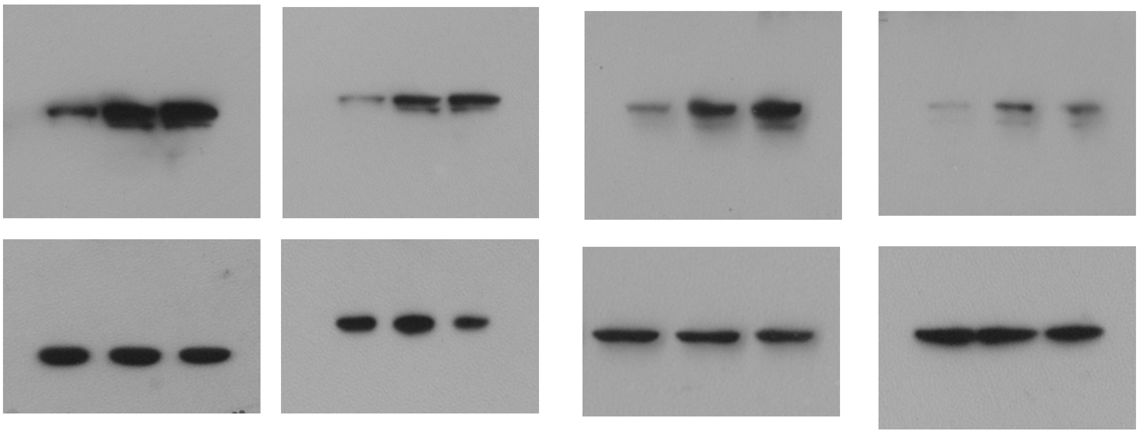

Supplementary Figure 7. **Full-length blots of Figure 4 (a) and (h) in the main text.**

Figure 4 (a)

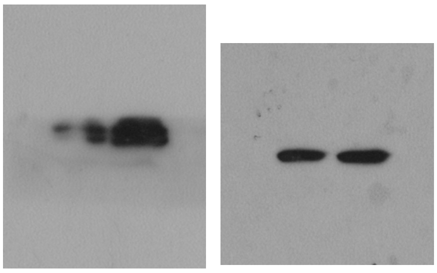

Figure 4 (f)

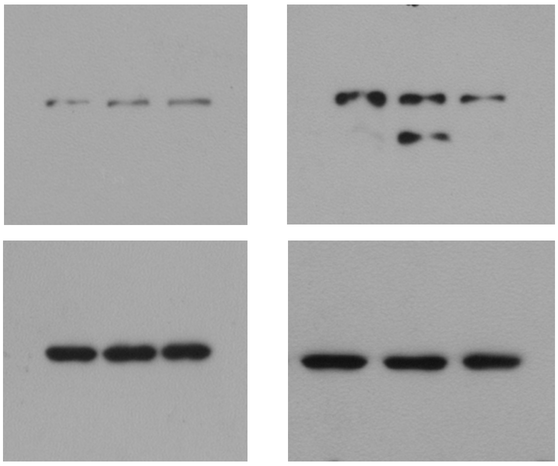

Supplementary Figure 8. **Full-length blots of Figure 5 (d) in the main text.**

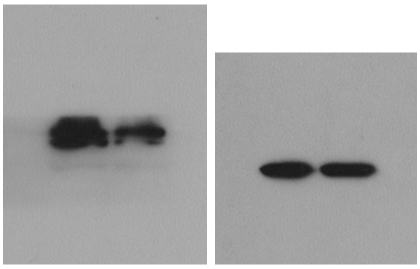

Supplementary Table 1. **Primer sequences for qPCR analysis.**

**Gene name**	**Forward primer (5'- 3')**	**Reverse primer (5'-3')**
Trpm1	GCACCCTGGGCAAGTATGG	ACGTTAGGACCACCTTCTACC
Axin2	AACCTATGCCCGTTTCCTCTA	GAGTGTAAAGACTTGGTCCACC
T (brachyury)	ACAACCACCGCTGGAAATATG	CTCTCACGATGTGAATCCGAG
Nanog	CACCCACCCATGCTAGTCTT	ACCCTCAAACTCCTGGTCCT
Tbx3	AGATCCGGTTATCCCTGGGAC	CAGCAGCCCCCACTAACTG
Tfcp2l1	CAGCCCGAACACTACAACCAG	CAGCCGGATTTCATACGACTG
Neurod1	ATGACCAAATCATACAGCGAGAG	TCTGCCTCGTGTTCCTCGT
Otx2	GAATCCAGGGTGCAGGTATGG	CTGAACTCACTTCCCGAGCTG
Id3	AGCTCACTCCGGAACTTGTG	ATCGAAGCTCATCCATGCCC
Dnmt3l	CACCCCTTGTTTGAGGGAGG	ATGGTGCAGTAACTCTGGTGT
Dnmt3a	GATGAGCCTGAGTATGAGGATGG	CAAGACACAATTCGGCCTGG
Fos	GTGAAGACCGTGTCAGGAGG	GATCTGTCTCCGCTTGGAGT
Junb	TCACGACGACTCTTACGCAG	CCTTGAGACCCCGATAGGGA
Socs3	CCCTTGCAGTTCTAAGTTCAACA	ACCTTTGACAAGCGGACTCTC
Myc	TCGCCCAAATCCTGTACCTC	GACCTCTTGGCAGGGGTTTG
Drosha	TCTGTAGAGACTGTGAATCCTGC	GCATCTCGATGCGTCCTGTA
pri-mir181a-2	ACCCAACAGCAGTGGTCCTTA	AGCCTACTCCTTTGCTTCAGG
pri-mir181b-2	GAAGAAGAGCCAGGAGATAGA	ATAAAAGATTGAGGTCCCACCTG
pri-mir-211	ACCTGTGGGCTTCCCTTTGTC	TCCAGAAGCAGAGAAGAACTTGC
Pri-miR-302	CTGTGGGTTTGCTCTTCTGTTTT	GAGACAGAAAGCATTCCCATGTT
Gapdh	GTGTTCCTACCCCCAATGTGT	ATTGTCATACCAGGAAATGAGCTT
miR-302a-5p	GGACTTAAACGTGGTTGT	The reverse primer for qPCR experiments of miRNAs is a universal primer provided by the miScript II RT Kit (QIAGEN).
miR-302b-5p	GGGACTTTAACATGGGAATG	
miR-302c-5p	GGGCTTTAACATGGGGTT	
miR-302d-5p	GGGACTTTAACATGGAGGC	
miR-130b-5p	GGACTCTTTCCCTGTTGCACTACT	
miR-134-5p	TGTGACTGGTTGACCAGAGGG	
miR-181a-5p	AACATTCAACGCTGTCGGTGAGT	

miR-181b-5p	AACATTCATTGCTGTCGGTGGGT	
miR-200c-3p	TAATACTGCCGGGTAATGATGGA	
miR-290-5p	ACTCAAACTATGGGGGCAC	
miR-106a-3p	ACTGCAGTGCCAGCACTTCTTAC	
miR-106b-3p	CCGCACTGTGGGTACTTGCT	
miR-93-5p	CAAAGTGCTGTTCGTGCAGG	
miR-21-5p	GGTAGCTTATCAGACTGATGTTGA	
miR-296-5p	AGGGCCCCCCCTCAATCCTGT	
miR-34a-5p	TGGCAGTGTCTTAGCTGG	
miR-211-3p	GCAAGGACAGCAAAGGGGGGC	
miR-34b-3p	AATCACTAACTCCACTGCCATC	
miR-470-5p	TTCTTGGACTGGCACTGGTG	
Pre-miR-302a	GGTTGTACTTGCTTTAGACCTAAG	
Pre-miR-302b	GAATGCTTTCTGTCTCATCGAAG	
Pre-miR-302c	ATGGGGTTACCTGCTGTGTTAAAC	
Pre-miR-302d	CATGGAGGCACTTGCTGTGCATTTA	
Pre-miR-181a-2	GTCGGTGAGTTTGGGATTCAAAAAC	
Pre-miR-181b-2	GCTGTCGGTGGGTTTGAATGTCAAC	
Rnu6	TGCGCAAGGATGACACGC	

Supplementary Figure Legend

Supplementary Figure 1. Experimental scheme for sample preparation of small RNA deep-sequencing and microarray analysis. J1 mESCs cultured in LIF containing media were treated with 1 μM BIO, 3 μM CHIR or equal volume of DMSO (control) for 24 h, and then the total RNA were extracted, qualified RNA were analyzed by microarray gene expression profiling and small RNA deep-sequencing to identify differentially expressed mRNAs and miRNAs.
